# Dangerous Receipts

**Published:** 1858-12

**Authors:** 


### DANGEROUS RECEIPTS.

NINE TENTHS of all newspaper receipts, in reference to health and disease, are either untrue, useless, or pernicious; hence, the whole of them, in order to be on the safe side, should be discarded, unless the name of a physician, or that of some respectable medical journal is attached. Editors of newspapers, from their obliging disposition, are often imposed upon by some weak or half-observant correspondent or subscriber. Scarce a day passes that we do not see these mischievous items.

A named article may be given for a named disease or symptom, and that symptom may disappear after it; and yet the remedy may be of no more value than a thimbleful of dust—for two reasons: First, the symptom in question may have disappeared of itself, as millions of symptoms have done, without any means whatever. The practice of educated physicians is, not to speak of a remedy as valuable for one, or half a dozen times, but for many, extending generally through years. Second, to know whether the remedy is applicable, we must know if all the conditions of the person about to take it are precisely alike those of the one who is reported to have been benefitted. Many persons have taken a "good dose of salts" for a cold, and soon got well. But the symptoms of a common cold are sometimes the symptoms of a dangerous malady. Last year, the lady proprietor of a female school in this city, observed one of her pupils to be unwell, and noticing a tendency to sneeze, running at the nose, a snuffling, and a fulness of look about the eyes and face, she thought it was a cold, and gave therefor the old-fashioned remedy, which she had known in childhood—a dose of salts—and the pupil, the only child of wealthy parents in a distant State, died next day. Why? The disease was scarlet fever. The salts acted on the bowels: this so weakened the system, that it had not strength enough left to throw the disease out on the surface. The man or woman—not a physician—is a dolt, who gives, takes, or advises any medicine whatever, for any symptom whatever, when a physician can be had within fifteen minutes. And such are not unfrequently brought up all standing by a palpable murder, to embitter the remainder of their days, as in the case we have just named. No reasoning, no philanthropy, no money, no time, will ever wipe out the remorse from that woman's mind. The purity of her intention can never atone for the impudent folly of her act, or restore that only daughter to hearts made desolate by her sacrifice.

